# Uncoupling of Glomerular IgA Deposition and Disease Progression in Alymphoplasia Mice with IgA Nephropathy

**DOI:** 10.1371/journal.pone.0095365

**Published:** 2014-04-17

**Authors:** Masashi Aizawa, Yusuke Suzuki, Hitoshi Suzuki, Huihua Pang, Masao Kihara, Junichiro Nakata, Kenji Yamaji, Satoshi Horikoshi, Yasuhiko Tomino

**Affiliations:** Division of Nephrology, Department of Internal Medicine, Juntendo University Faculty of Medicine, Tokyo, Japan; University of Tokyo, Japan

## Abstract

Previous clinical and experimental studies have indicated that cells responsible for IgA nephropathy (IgAN), at least in part, are localized in bone marrow (BM). Indeed, we have demonstrated that murine IgAN can be experimentally reconstituted by bone marrow transplantation (BMT) from IgAN prone mice in not only normal mice, but also in alymphoplasia mice (*aly/aly*) independent of IgA^+^ cells homing to mucosa or secondary lymphoid tissues. The objective of the present study was to further assess whether secondary lymph nodes (LN) contribute to the progression of this disease. BM cells from the several lines of IgAN prone mice were transplanted into *aly/aly* and wild-type mice (B6). Although the transplanted *aly/aly* showed the same degree of mesangial IgA and IgG deposition and the same serum elevation levels of IgA and IgA-IgG immune-complexes (IC) as B6, even in extent, the progression of glomerular injury was observed only in B6. This uncoupling in *aly/aly* was associated with a lack of CD4^+^ T cells and macrophage infiltration, although phlogogenic capacity to nephritogenic IC of renal resident cells was identical between both recipients. It is suggested that secondary LN may be required for the full progression of IgAN after nephritogenic IgA and IgA/IgG IC deposition.

## Introduction

IgA nephropathy (IgAN) is the most common form of primary glomerulonephritis and exhibits mesangial IgA and IgG codeposition [1]. However, the mechanisms of mesangial IgA deposition and the origin of nephritogenic IgA remain unclear. Many studies have convincingly suggested the involvement of dysregulation in the mucosal immune system. Mesangial IgA and an increased serum IgA fraction in patients with IgAN are predominantly polymeric IgA1 (pIgA1) [2], [3]. Several studies have shown the numbers of IgA1^+^ plasma cells are increased in the bone marrow (BM) of patients with IgAN [4], [5]. Moreover, bone marrow transplantation (BMT) or peripheral blood stem cell transplantation in patients with leukemia and IgAN has resulted in a remission of leukemia as well as IgAN [6], [7]. These findings suggest that the cells responsible for producing pathogenic IgA1 may exist, at least in part, in the BM of IgAN patients.

The ddY mouse is known as a spontaneous IgAN prone mouse [8], although the incidence of their IgAN is highly variable [8], [9]. We found that the mice could be divided into the following three groups through a longitudinal histological analysis: early onset, late onset, and a quiescent group [10]. A genome-wide association study between the early onset and quiescent mice showed that one of the susceptibility loci of murine IgAN is syntenic to the susceptibility loci of human IgAN [10]–[12]. These findings indicated that this murine IgAN might be, at least in part, under the same genetic regulation as in human IgAN. Moreover, IgAN onset ddY mice exhibited elevated levels of serum IgA-containing immune-complexes (IC) and mesangial IgA and IgG co-deposition, as observed in human IgAN [13]. Nasal challenge with unmethylated CpG dinucleotides (CpG DNA), by which bacteria and viruses are distinguished and the Toll-like receptor (TLR)-9 is activated, worsened glomerular injury in the onset ddY mice and was associated with greater mesangial IgA deposition, higher serum IgA levels, and strong Th1 polarization [14]. We succeeded in generating several lines of a “grouped ddY mouse” that are a mouse model of IgAN with 100% onset after crossbreeding early onset mice for more than 20 generations [15]. Thus, it is suggested that the grouped ddY mouse can be a useful model for studying the pathogenic mechanisms of IgAN.

We also reported that BMT from the onset IgAN prone mice induced IgAN and that the serum levels of IgA-IgG IC were significantly correlated with the severity of glomerular injury [13], [16], [17]. However, the underlying mechanism by which the BM cells (BMC) induce IgAN remains unclear. Moreover, it was still unclear whether BMC directly produce nephritogenic IgA or require additional encounters with certain antigens in lymphoid tissues. To answer this question, we performed BMT and the adoptive transfer of cells from Peyer’s patches (PP) from IgAN prone mice and alymphoplasia mice (*aly/aly*) lacking PP, all lymph nodes (LN), and immunoglobulin producing cells due to a point mutation in the NF-κB-inducing kinase (NIK) gene on a C57BL/6 (B6) background [18]. A recent study demonstrated that the injection of PP cells of control mice into *aly/aly* mice induced both the migration of PP cells into the lamina propria (LP) and the generation of IgA^+^ plasma cells, thus rescuing gut IgA. On the other hand, the transplantation of BMC from normal control mice into *aly/aly* mice failed to rescue gut IgA, in spite of a recovery of serum IgA and the presence of IgA^+^ B cells and plasma cells [19]. Indeed, we observed that BMC induced glomerular IgA deposition independently of homing to the mucosa and secondary lymphoid tissues in the murine IgAN. Furthermore, BM may be a major reservoir of cells producing glomerular IgA. However, BMC could not induce the full progression of glomerular injury after IgA deposition in *aly/aly* mice. The objective of the present study using *aly/aly* mice was to further assess how secondary LN contribute to the progression of murine IgAN.

## Materials and Methods

### Ethics Statement

All animal studies were approved by the Ethics Review Committee for Animal Experimentation of the Juntendo University Faculty of Medicine. Animal procedures were conducted in compliance with National Institutes of Health Guidelines.

### Mice

Two lines (A and B) of grouped ddY mice [10], [15], [17], aly/NSCJcl-aly (*aly/aly*) mice (CLEA Japan, Tokyo, Japan) with B6 background, and wild type C57/BL6 (B6) mice (SLC Japan) were maintained in the animal facility of Juntendo University in a specific-pathogen-free (SPF) room. For BMT, all mice of both lines of grouped ddY mice showed the disease phenotypes of IgAN from 4–6 weeks of age.

### Bone Marrow Transplantation (BMT)

We analyzed the histological changes of the kidneys and measured the levels of serum immunoglobulins. The procedure for murine BMT was previously described in detail [13]. In brief, BMC were harvested from the tibia, femur and humerus of the IgAN onset ddY mice (A and B) [15] at 18–20 weeks of age under sterile conditions. Red blood cells were depleted from the collected BMC. Female *aly/aly* mice at 8–9 weeks of age and the same-aged B6 mice were used as recipients. Then, 1×10^7^ BMC were injected into the tail vein of irradiated recipient mice at 700 rad. Transplanted mice were housed under SPF conditions. Transplanted *aly/aly* and control B6 mice were sacrificed at 12 and 24 weeks after BMT. We examined the histology of the kidneys and conducted a FACS analysis of spleen cells and BMC. The levels of serum immunoglobulins, IC, and urinary albumin were measured.

### Histological Examination

For light microscopy, the kidney specimens were fixed in 20% neutral phosphate-buffered formalin, embedded in paraffin and then stained with periodic acid-Schiff (PAS) reagents. All specimens of BM transplanted mice were analyzed quantitatively to determine the percentages of glomeruli with (*1*) segmental and global sclerosis, (*2*) mesangial cell proliferation and cellular infiltration, and/or (*3*) an increase in the mesangial matrix. All specimens were scored semiquantitatively for the percentage of the aforementioned lesioned glomeruli (score 0, 0%; score 1, 1 to 19%; score 2, 20 to 39%; score 3, 40 to 59%; score 4, 60 to 79%; and score 5, >80% of all glomeruli) [20]. Glomerular polymorphonuclear cells (PMNs) of the anti-glomerular basement membrane (GBM) glomerulonephritis (GN) mice were counted in 3 µm sections that had undergone a PAS staining. The numbers of PMNs were determined in at least 20 glomeruli per mouse. For immunohistochemistry, the tissue samples were frozen in an OCT compound. Sections 5 µm thick were fixed in 4% paraformaldehyde. The following reagents were used for staining: FITC- rat anti-mouse IgA, FITC- rat anti-mouse IgG, FITC- rat anti-CD4 (BD Biosciences, Pharmingen; San Diego, CA), FITC- anti-mouse macrophages (F4/80) (Funakoshi, Tokyo, Japan), and rhodamine-conjugated goat anti-mouse IgA (Santa Cruz Biotechnology, Santa Cruz, CA) antisera. For electron microscopy, the specimens were fixed in phosphate-buffered glutaraldehyde for 2 hours, postfixed in 2% osmium tetroxide for 2 hr, and then embedded in Epon resin after dehydration. Ultrathin sections were sliced at 70 nm, stained with 4% uranyl acetate and lead citrate, and then examined under an electron microscope (Hitachi 7100, Tokyo, Japan).

### Serum and Urinary Examination

Blood samples were obtained from the orbital venous plexus using capillary tubes. The serum samples were stored at −80°C prior to use. The serum levels of IgG and IgA were measured by single radioimmunodiffusion (SRL, Tokyo, Japan). Level of serum IgA/IgG IC was measured by sandwich ELISA according to a method described previously [13]. In brief, an ELISA plate was coated with 5 µg/ml rat anti-mouse-IgG antisera (Pharmingen) at 4°C overnight. Coated plates were blocked with 2% BSA in PBS, and serum samples were incubated at 4°C overnight. After incubation with HRP-conjugated goat anti-mouse IgA antisera (ZYMED Laboratories, San Francisco, CA) at room temperature for 3 hr, the reaction was developed with a tetramethylbenzidine substrate reagent (BD Biosciences, San Jose, CA), and the absorbance was measured at 540 nm. Data were expressed relative to the value of BALB/c mice. Urinary albumin was measured using an ELISA kit (Albuwell; Exocell, Philadelphia, PA). Serum and urine samples from BALB/c and B6 mice were used as controls.

### Monocyte Chemotactic Protein (MCP)-1 Production in Primary Mesangial Cells

Mouse mesangial cells (MC) were cultured from outgrowths of glomeruli harvested as described previously [21]. In brief, *aly/aly* and control B6 mice were sacrificed by decapitation, and their kidneys were removed. After removal from the renal capsule, the kidneys were minced and treated with collagenase. The components of glomeruli were separated by repeated 30–50% Percoll gradient centrifugation and were cultured in an RPMI-1640 medium supplemented with 20% FCS, 100 U/ml penicillin, 100 µg/ml streptomycin, insulin, and transferine in a 5% CO_2_ environment at 37°C. On the 21st day of culture, MC were trypsinized and subcultured for 3 days in 6-well plates.

Heat-aggregated IgA was generated through the incubation of mouse myeloma IgA (Cappel, MP Biomedicals LLC., Solon, OH) at 63°C for 150 min [22]. After centrifugation, insoluble aggregates were discarded. After washing 3 times with a serum-free culture medium, subconfluent MC in 6-well plates were incubated with 200 µg/ml of heat-aggregated IgA in a 1 ml RPMI-1640 medium with 0.5% FCS. Cell-free supernatants were collected after 6, 12, and 24 hr of incubation and stored at −80°C until assayed for MCP-1 (n = 3, each). MCP-1 concentrations in the supernatants were measured using an OptEIA Mouse MCP-1 Set (Pharmingen).

### Anti-glomerular Basement Membrane (GBM) Glomerulonephritis (GN) Induction

The method for preparation of nephrotoxic serum (NTS) was previously described [23]. In brief, NTS was kindly provided by Kyowa Hakko Kogyo Co. Ltd. (Mishima, Japan). Anti-GBM GN was induced by an intravenous injection of NTS through the tail vain in *aly/aly* mice (n = 3) and control B6 mice (n = 3). We collected the urine of the mice and sacrificed them 6 hours after the NTS injection. We examined the degree of polymorphonuclear cells (PMN) influx and the histology of the kidneys.

### Statistical Analysis

Data were analyzed by using the paired Student’s *t* test. *P* values of less than 0.05 were considered as significant. All statistical analyses were performed using StatView 5.0 software (Abacus Concepts Inc., Berkeley, CA).

## Results

### Reconstitution of Glomerular IgA Deposition by BMC from the IgAN Prone Mice

**Figure 1:**
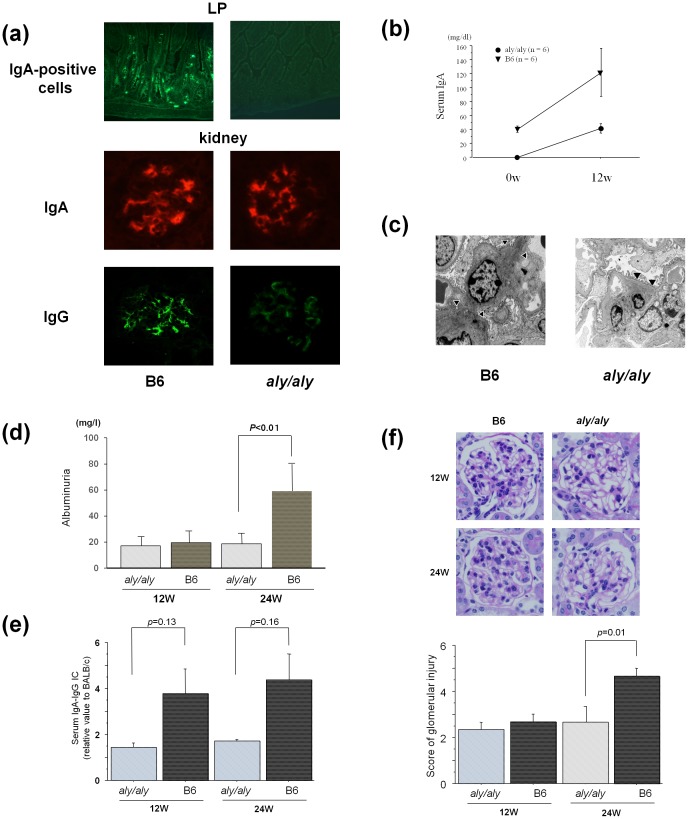
Reconstitution of IgA nephropathy by BM transplantation from ddY mice in *aly/aly* and B6 mice. BM transplantation (BMT) from IgAN onset ddY mice (line A) (N = 24) reconstituted glomerular IgA (a) with serum elevation of IgA (b) in *aly/aly* and WT control B6 mice (*aly/aly* and B6 at 12 and 24 w, n = 6, each) independently of homing of IgA-positive cells into lamina propria (LP). Clear glomerular IgG deposition was found in B6, but not in *aly/aly* mice. Although electron-dense deposits (arrow head) were detected in *aly/aly* mice at 24 w after BMT as well as B6 mice (c), progression of urinary protein (d) and glomerular lesions (f) were absent in *aly/aly* mice in association with the lack of serum elevation of IgA-IgG immune complex (IC) (e). Data are presented as mean ± SD.

The BMT from IgAN onset grouped ddY mice (line A) reconstituted glomerular IgA deposition (Figure 1a), with an increase of serum IgA (Figure 1b) and the number of IgA^+^B220^−^CD138^+^ plasma cells (data not shown), in *aly/aly* mice at 12 weeks as well as in B6 mice (n = 6, each). However, the migration of IgA positive cells to the lamina propria was not observed in *aly/aly* mice at 12 weeks after BMT (Figure 1a). Paramesangial electron-dense deposits were confirmed by electron microscopy in the transplanted *aly/aly* mice (Figure 1c). These data suggest that BMC can directly reconstitute glomerular IgA deposition and the homing process in the LP is not required as reported in our previous study [24].

### Uncoupling of Glomerular IgA Deposition and Disease Progression in Transplanted *Aly/Aly* Mice from IgAN Prone Mice (Line A)

Reconstituted glomerular IgA deposition, the level of albuminuria, and the glomerular injury score were not significantly different between *aly/aly* and B6 recipients at 12 weeks after the BMT (Figure 1d). The progression of albuminuria and glomerular injury with an increase of IgA-IgG IC (Figure 1e), mesangial cell proliferation, and mesangial matrix expansion were observed in the B6 recipient mice after 24 weeks (Figures 1f). On the other hand, although the transplanted *aly/aly* mice showed glomerular IgA deposition, they did not show either an increase of IgA-IgG IC and clear glomerular injury or a progression of albuminuria at 24 week after BMT (Figures 1a, c–f).

### No Difference of Phlogogenic Capacity to Nephritogenic Antibody of Renal Resident Cells in both *Aly/Aly* and B6 Mice

**Figure 2:**
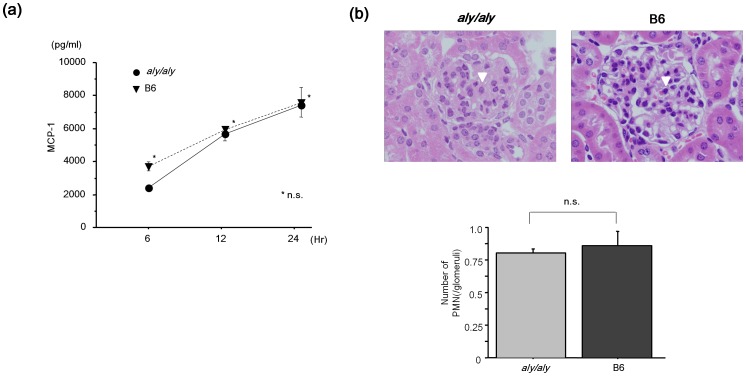
Identical phlogogenic capacity of renal resident cells in *aly/aly* and B6 mice. (a) To confirm the nephritogenic capacity to of renal resident cells in *aly/aly* and B6 mice, chemokine expressions, such as MCP-1, were examined with mesangial cells from both mice under stimulation with IgA IC. MCP-1 concentrations in supernatants of both mice were not significantly different (n = 3, each). (b) Next to confirm phlogonenic capacity of aly/aly mice, anti-GBM glomerulonephritis was induced in *aly/aly* and B6 mice (n = 3 each). An influx of PMN (arrow head) was shown in the glomerulus of both mice. The number of PMN in the glomerulus of *aly/aly* and B6 mice were not significantly different. Data are presented as mean ± SD.

To approach underlying mechanism for the uncoupling of IgA deposition and progression of glomerular injury in *aly/aly* mice, we first checked the phlogogenic capacity to nephritogenic antibody of renal resident cells in both mice with or without the NIK mutation. MC-derived chemokine, MCP-1, mainly depending on NF-κB activation, is known as a key mediator for the progression of IC-induced glomerulonephritis (24). The primary MC from *aly/aly* and B6 mice was stimulated by heat-aggregated IgA and the MCP-1 production was evaluated, in order to exclude the possibility that an NIK mutation in the intrinsic renal cells of *aly/aly* mice may directly influence *in vivo* findings. MCP-1 production was increased in a time-dependent manner in both *aly/aly* and B6 mice (Figure 2a). In addition, these levels were not significantly different in both mice, suggesting that major chemokine production by MC after IgA deposition may be independent of the NIK mutation in *aly/aly* mice.

### No Difference of the Response of Anti-GBM GN in *Aly/Aly* Mice and B6 Mice

To further examine the phlogogenic capacity of *aly/aly* mice, we next used a different acute *in vivo* model. Anti-GBM GN was induced in *aly/aly* and B6 mice. In both mice, we observed a glomerular PMN influx, and the number of PMN in these glomeruli was not significantly different between *aly/aly* and B6 mice (Figure 2b). Thus, the acute phlogogenic capacity of *aly/aly* mice is not different from B6 mice.

### The Uncoupling of Glomerular IgA Deposition and Disease Progression in Transplanted *Aly/Aly* Mice from the IgAN Prone Mice

**Figure 3:**
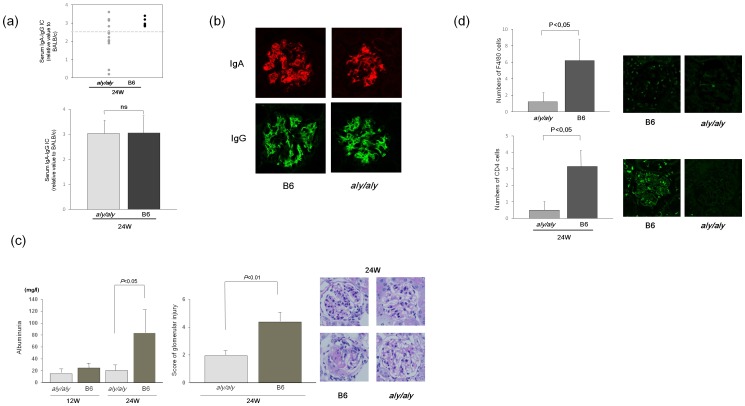
Uncoupling of glomerular IgA and disease progression in transplanted *aly/aly* mice was independent of IgA-IgG IC formation and deposition. To further assess underlying mechanisms in the lack of the disease progression of IgAN in *aly/aly* mice, BM cells from a different line of poor-prognosis IgAN prone (line B) were transplanted in both mice. Although the serum levels of IgA-IgG IC were variable, some of the transplanted *aly/aly* mice (n = 5) showed similar amounts of IC (>2.5) to B6 mice (n = 5) (a). These selected *aly/aly* mice showed clear glomerular deposition of not only IgA but also IgG at 24 w, as seen in B6 mice (b). However, the progression of proteinuria and glomerular lesions was absent (c). This was associated with lack of F4/80 positive macrophages and CD4^+^ T cells (d). Data are presented as mean ± SD.

In BMT from line A, IgG co-deposition at 24 weeks of age in *aly/aly* mice was less than that of B6 mice in association with lower levels of serum IgA-IgG IC (Figure 1). To assess whether lower IgG IC formation and its glomerular deposition may result in an uncoupling in *aly/aly* mice, we transplanted BM from grouped ddY mice line B, which show poorer prognosis in association with higher IC formation than line A [15]. Serum levels of IgA-IgG IC formation at 24 weeks in *aly/aly* mice transplanted from line B were variable but higher than those from line A (Figure 1e and 2a). Although they were lower than those in the control B6 on average, some of *aly/aly* recipients from line B (more than 2.5; N = 5) (Figure 3a) showed similar levels of serum IgA-IgG IC and IgG co-deposition as the control (Figure 3b). However, even in the selected *aly/aly* mice (n = 5) that showed similar amounts of serum IgA-IgG IC and IgG codepositions as the B6 control (n = 5), the progression of renal damage, including albuminuria and an injury score, was absent (Figure 3c) as compared to BMT from line A. This is associated with the lack of F4/80 (macrophages) and CD4^+^ T cells infiltration (Figure 3d).

## Discussion

Several studies have shown that the number of IgA1^+^ plasma cells increased in the BM of patients with IgAN [4], [5] and that BMT or peripheral blood stem cell transplantation in patients with leukemia and IgAN resulted in a remission of the leukemia as well as the IgAN [6], [7], suggesting that the producing cells of nephritogenic IgA may be in the BM of IgAN. Indeed, we reported that BMT from onset IgAN-prone mice induced IgAN in healthy mice [13]. However, it is clinically well known that many IgAN patients show episodic macrohematuria coincident with mucosal infection, especially in the upper respiratory tract [26]–[28]. Mucosal vaccination studies revealed abnormal immune responses in the mucosa of patients with IgAN [29], [30]. Importantly, there is increasing evidence that tonsillectomy has a favorable effect on the long-term renal survival of IgAN patients [31]–[33]. We reported that TLR9 activation by nasal challenge with CpG DNA worsened glomerular injury in ddY mice and was associated with strong Th1 polarization [14], [34], [35]. In this regard, several reports support the idea that exogenous antigens via the respiratory tract or gut induce glomerular IgA deposition [14, 34, 36–38]. Therefore, these observations indicate that the mucosal immune response of IgAN patients might play a role in pathogenesis, and furthermore, responsible cells may be disseminated to not only the BM, but also to mucosa or systemic lymphoid tissues [16].

Our previous study demonstrated that BMC of onset ddY mice could reconstitute glomerular IgA deposition in *aly/aly* mice lacking PP, all LN and without migration of IgA^+^ cells to LP [24]. These findings suggested that the mechanism of nephritogenic IgA production might not require other immune responses or the maturation of BMC in secondary lymphoid tissues and mucosa, at least in murine IgAN. In addition, we first showed that the homing process of IgA^+^ cells into the mucosa is not necessary for nephritogenic IgA production. On the other hand, PP transfer of onset ddY mice could not reconstitute glomerular IgA deposition [24]. The findings suggest that producing cells of nephritogenic IgA do not constitute PP of onset ddY mice. Accordingly, we hypothesized that the mucosa may be the major initial priming site of cells responsible for nephritogenic IgA production, while BM may be a major reservoir of these cells, presumably memory type cells, and a major site of nephritogenic IgA production [39].

This raises the question as to whether an NIK mutation in intrinsic renal cells of *aly/aly* mice may protect renal injuries followed by mesangial IgA deposition. Several studies [25], [40] have clearly demonstrated that the activation of NF-κB, known as a key inflammatory transcriptional factor in renal resident cells, is a major process in the progression of IC-mediated glomerulonephritis. MCP-1 has an important role in the development of renal injuries [41], [42]. Emerging evidence has indicated that the upregulation of glomerular MCP-1 during the proliferative phase of glomerulonephritis is, at least in part, due to the NF-κB dependent signaling pathway [43], [44]. Levels of MCP-1 produced by mesangial cells stimulated with heat-aggregated IgA in *aly/aly* mice did not differ from those in B6 mice. It is suggested that the NIK mutation, at least in mesangial cells, may not affect major chemokine production by MC and the progression of IgAN, such as albuminuria, proliferation of mesangial cells, and the expansion of mesangial matrix, followed by mesangial IgA deposition in transplanted *aly/aly* mice. To further confirm this idea, we checked the phlogogenic capacity of *aly/aly* mice in a different *in vivo* model. The anti-GBM GN model is often used to investigate acute inflammatory cascade leading to immunologically-mediated GN [45]. Anti-GBM GN consists of two distinct phases, namely a heterologous phase, resulting from a rapid binding of the injected heterologous anti-GBM antibodies, and autologous phase that is induced when the recipient’s cells react with its own antibodies against the heterologous immunoglobulin previously fixed at the GBM. The severity of renal damage in the heterologus phase is known to be dependent on the glomerular influx of PMN [21], [45]. Our study shows that a normal influx of PMNs as heterologus phase of the anti-GBM GN is also induced in *aly/aly* and B6 mice. Although we may need to further analyze the effect of the NIK mutation in kidney disease, these results show that the NIK mutation is not likely to have an impact in the absence of an influx of PMN in the acute immune response after antibody deposition and glomerular injuries, representative of a proliferation of mesangial cells. In addition, previous studies showed that present protocol of BMT functionally exchanged most of leukocytes in recipients from original to transferred BM-derived leukocytes [14], [15], [46], [47]. Therefore, the uncoupling of glomerular IgA deposition and disease progression in the transplanted *aly/aly* mice from onset ddY mice is not influenced by the NIK mutation.

In general, the mild glomerular deposition of IgA is not always accompanied by proteinuria and hematuria. Moreover, in so-called secondary IgAN [48], [49] observed in patients with liver cirrhosis [50], portal systemic shunts [51], dermatitis herpetiformis [52], celiac disease [53], and chronic inflammatory disease of the lung [54], glomerular IgA deposition without obvious glomerular lesions [48] is frequently observed. Meanwhile, many studies have demonstrated emerging evidence that mesangial IgA in IgAN is exclusively the aberrantly glycosylated IgA1 subclass, in particular, galactose-deficient in the hinge region of IgA1 (GdIgA) [55], [56]. However, Gharavi et. al. reported that a high serum level of GdIgA1 was determined in a cohort of not only IgAN patients but also their relatives without nephropathy [57]. This finding indicates that other mechanisms besides GdIgA1 are required for the full progression of IgAN.

Although the functional significance of the altered IgA is still obscure, Tomana et al. reported that the level of circulatory autoantibodies against aberrantly glycosylated IgA1 was elevated in patients with IgAN [58]. Recently, Suzuki et al. reported that the development of a dot-blot assay using Gal-deficient IgA1 as an antigen and GalNAc-specific recombinant human IgG from an IgAN patient as a standard permitted accurate analysis of the levels of glycan-specific IgG *in sera* with high specificity and sensitivity and the serum levels of this IgG, which is specific for Gal-deficient IgA1, correlated with the clinical parameter of proteinuria [59]. It suggests that aberrantly glycosylated IgA1 is recognized as an endogeneous antigen and forms IC with circulatory autoantibodies in human IgAN [59]. Such multi-hit processes are now hypothesized in IgAN [60] and may decide the heterogeneity of IgAN [61].

This picture is also seen in murine IgAN. The severity of murine IgAN is linked to to the serum levels of IgA-IgG IC but not IgA [14]. On the other hand, the progression of murine IgAN may also link to abberant glycosylation of IgA as seen human IgAN [15]. We recently reported that polymerization or IC formation, presumably due to aberrantly glycosylated IgA, is important for the complement activation in murine IgAN [17]. Furthermore, mice lacking β-1,4-galactosyltransferase-I, which transfers galactose to the terminal N-acetylglucosamine of *N*-linked glycans in a β-1,4 linkage, showed a complete absence of β4 galactosylation on the *N*-glycans of mouse IgA and subsequently spontaneous development of IgAN with increased serum polymeric IgA levels [62]. These findings suggest that the aberrant glycosylation of serum IgA, presumably as an endogeneous antigen, may be involved in the induction of IgAN, even in mice. Therefore, we initially speculated that the transplanted *aly/aly* mice from line A could not form IC containing antibodies recognized as an endogeneous antigen because the mice are lacking secondary lymphoid tissues and because the mice did not induce sufficient IgA/IgG co-deposition and glomerular injury.

To confirm this idea, we further did BMT with different clones of grouped ddY mice line B, which we recently established by cross-breeding [15]. This prone mouse shows a more severe prognosis of murine IgAN with an increase of serum levels of IgA-IgG IC [13], [17] that may be partly due to the pattern of glycosylation in IgA. In this additional model, we indeed found a greater increase of IgA-IgG IC and clear IgG deposition. This finding suggests that secondary lymphoid tissues may be not required for at least the circulating IC formation. However, even though we selected *aly/aly* recipients showing similar amounts of glomerular IgG co-deposition, full progression of murine IgAN in *aly/aly* recipients was absent, suggesting other possible underlying mechanisms. Interestingly, this uncoupling in *aly/aly* mice is linked to a lack of CD4^+^ T cells and macrophage infiltration. It is recently known that the renal prognosis of IC-mediated glomerulonephritis, including IgAN and lupus nephritis, is significantly associated with the degree of T cell infiltration [63]–[65]. Accordingly, antigen presentation in the renal LN and the subsequent activation of cellular immunity may be involved in chronic and full progression of murine IgAN after the nephritogenic IgA and IC deposition in glomerular mesangial areas.

The uncoupling of glomerular IgA/IC deposition and disease progression associated with a lack of CD4 and macrophage infiltration in *aly/aly* mice indicates that secondary LN, presumably renal LN, may be required for the activation of cellular immunity and chronic progression of this disease. Multi-hit theory in the pathogenesis of IgAN [60] should involve the mechanisms in renal LN after the deposition of nephritogenic IgA/IC.
